# Endovascular Therapy for Acute Ischemic Stroke of Intracranial Atherosclerotic Origin—Neuroimaging Perspectives

**DOI:** 10.3389/fneur.2019.00269

**Published:** 2019-03-20

**Authors:** Oh Young Bang, Byung Moon Kim, Woo-Keun Seo, Pyoung Jeon

**Affiliations:** ^1^Department of Neurology, Samsung Medical Center, Sungkyunkwan University School of Medicine, Seoul, South Korea; ^2^Department of Radiology, Severance Hospital Stroke Center, Yonsei University College of Medicine, Seoul, South Korea; ^3^Department of Radiology, Samsung Medical Center, Sungkyunkwan University School of Medicine, Seoul, South Korea

**Keywords:** atherosclerosis, neuroimage, endovascular therapy, acute ischemic stroke, intracranial

## Abstract

Large vessel occlusion (LVO) due to intracranial atherosclerosis (ICAS) is a common cause of acute ischemic stroke (AIS) in Asians. Endovascular therapy (EVT) has been established as the mainstay of treatment in patients with AIS and LVO. However, only a few patients of Asian descent with ICAS-related LVO (ICAS-LVO) were included in recent randomized controlled trials of EVT for AIS. Therefore, the findings of these trials cannot be directly applied to Asian patients with ICAS-LVO. In embolic LVO due to thrombus from the heart or a more proximal vessel, rapid, and complete recanalization can be achieved in more than 70–80% of patients, and it is important to exclude patients with large cores. In contrast, patients with ICAS-LVO usually have favorable hemodynamic profiles (good collateral status, small core, and less severe perfusion deficit), but poor response to EVT (more rescue treatments and longer procedure times are required for successful recanalization due to higher rates of reocclusion). Patients with ICAS-LVO may have different anatomic (plaque, angioarchitecture), hemodynamic (collateral status), and pathophysiologic (thrombus composition) features on neuroimaging compared to patients with embolic LVO. In this review, we discuss these neuroimaging features, their clinical implications with respect to determination of EVT responses, and the need for development of specific EVT devices and procedures for patients with ICAS-LVO.

## Introduction

Large vessel occlusion (LVO), thought to originate from intracranial atherosclerosis (ICAS), is a common cause of acute ischemic stroke (AIS) in Asians (1). Embolic LVO due to thrombus from the heart or a more proximal vessel and ICAS-related LVO (ICAS-LVO) both show similar luminal changes and are treated with endovascular therapy (EVT) in acute settings. However, recent clinical studies suggest that treatment responses may differ between these two types of LVO (2–10). Patients with ICAS-LVO may have different anatomic (plaque, angioarchitecture), hemodynamic (collateral status), and pathophysiologic (thrombus composition) features on neuroimaging compared to patients with embolic LVO.

In this review, we discuss these features, their clinical implications with respect to determination of EVT responses, and the need for development of specific EVT devices and procedures for patients with ICAS-LVO.

## Search Strategy and Selection Criteria

We searched PubMed and ClinicalTrials.gov for articles published in English up to September 2018 using the following search terms: stroke, cerebrovascular disease, endovascular therapy, and intracranial stenosis. We also searched references from relevant articles and reviews. The final reference list was generated based on originality and relevance to this topic. We did not discuss individual imaging techniques or etiologies of non-atherosclerotic intracranial arterial disease in depth, since these topics are reviewed elsewhere (11–18).

## ICAS-LVO in Recent Randomized Controlled Trials of EVT for AIS

Phase III, randomized controlled trials (RCTs) conducted in 2015 demonstrated overwhelming evidence of the benefit of early window EVT for treatment of AIS with small core and LVO (19–23). More recently, the results of phase III RCTs of EVT in extended time windows showed significant and remarkable functional recovery after EVT compared to medical treatment in carefully selected patients (24, 25). In individual patient data meta-analyses of RCTs, the benefits of EVT were consistent in all prespecified subgroups of age, sex, initial stroke severity score, site of vessel occlusion, presence of tandem occlusion, extent of initial early ischemic changes on computed tomography (CT), intravenous tissue plasminogen activation (tPA), and onset-to-randomization time (26, 27). However, the type of LVO was not considered in the RCTs, and the number of patients with ICAS-LVO was small considering that only few Asian patients were enrolled in the 2 RCTs (20, 22).

The results of EVT in patients with ICAS-LVO are shown in Table 1. Recanalization failure, residual stenosis, and reocclusion were more frequently observed than embolic occlusion and rescue therapy with permanent stent placement or adjuvant antithrombotics are often required after EVT in ICAS-LVO patients (2–4, 9). Consequently, longer procedure times were required and higher complication rates and poorer long-term outcomes were reported after EVT in patients with ICAS-LVO than in those with embolic occlusion (5, 6, 8). Therefore, the results of the phase III RCTs of EVT cannot be directly applied to patients with ICAS-LVO.

**Table 1:** Summary of literature on angiographic features suggesting large vessel occlusion of suspected intracranial atherosclerotic origin and outcomes after endovascular therapy.

**References**	**Vascular territory**	**Diagnosis of ICAS-LVO**	**Main findings**	**Implications**
Baek et al. (2)	Any	Truncal-type occlusion	Reocclusion 77% of ICAS (*n* = 22) 5% of Embolic (*n* = 202)	Reocclusion was common and additional modalities are needed in ICAS
Baek et al. (3)	Carotid	Truncal-type occlusion	mTICI 2b-3 (with stentriever) 29% of ICAS (*n* = 56) 94% of Embolic (*n* = 262)	ICAS showed a low recanalization rate with strentriever and a similar rate with rescue therapy
Hwang et al. (4)	Any	Residual stenosis and tandem occlusion	Residual stenosis 100% of ICAS (*n* = 40) 28% Embolic (*n* = 123)	54% of patients with residual stenosis had ICAS
Al Kasab et al. (5)	Any	Fixed stenosis	Procedure time 99 min in ICAS (*n* = 36) 37 min in Embolic (*n* = 165)	Longer procedure time and poorer outcome in ICAS
Kim et al. (6)	V-B	Residual stenosis or reocclusion	Procedure time 96 min in ICAS (*n* = 19) 61 min in Embolic (*n* = 32)	Longer procedure time and poorer outcome in ICAS
Kang et al. (7)	Any	Fixed stenosis or aggravation after IA injection of vasodilator	mTICI 2b-3 in ICAS (*n* = 140) 96% in angioplasty/stent 94% in IA GP inhibitor	Both angioplasty/stenting and IA GP inhibitor are effective
Lee et al. (8)	Carotid	Residual stenosis >70%, or stenosis ≤ 70% with a tendency toward reocclusion and/or flow impairment during the procedure	mTICI 2b-3 76.8% in ICAS (*n* = 99) 79.6% in Embolic (*n* = 421)	ICAS showed similarly successful reperfusion rates but poorer functional outcome with EVT than embolic occlusion
Gascou et al. (9)	Any	Not specified	ICAS in 8 Embolic in 136	ICAS was associated with recanalization failure and higher rates of complication and mortality
Yang et al. (10)	Carotid	Fixed stenosis or retrospective analysis of the TOAST classification	Favorable outcome at 90 days in ICAS (*n* = 302) 48% in stentriever group 70% in angioplasty and/or stenting group	Angioplasty and/or stenting as first-line therapy may be superior to thrombectomy in ICAS

*ICAS, intracranial atherosclerosis; V-B, vertebrobasilar; mTICI, modified treatment in cerebral ischemia score; IA, intra-arterial; GP, glycoprotein IIb/IIIa; EVT, endovascular therapy; TOAST, Trial of Org 10172 in Acute Stroke Treatment*.

## Diagnosis of Suspected ICAS-LVO

**Figure 1:**
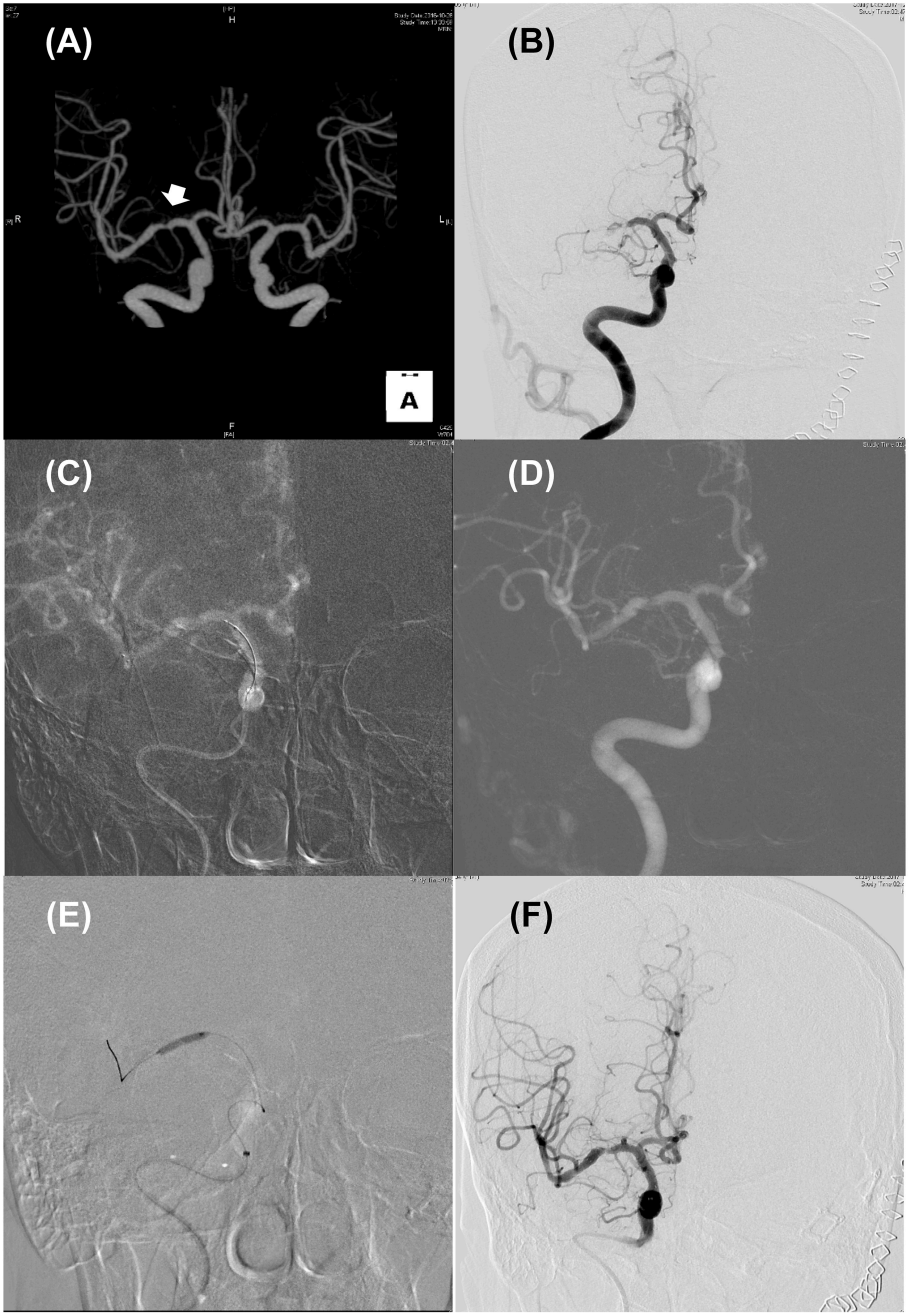
Illustrated case for the management of acute stroke due to intracranial atherosclerosis. **(A)** CT angiography performed 2 years ago revealed focal stenosis on right mid-MCA (Arrow). **(B)** Initial internal carotid angiography showed truncal-type occlusive lesion on right mid-MCA with minimal blood flow across the occlusive lesion. **(C,D)** Roadmap images during solitaire stent (4 × 20 mm) placement **(C)** and after retrieval **(D)**. Pre-existing stenotic lesion still be seen. **(E)** Balloon angioplasty using Gateway TPA balloon (2 × 15 mm; Boston scientific) was performed. **(F)** Delayed carotid angiography 30 min after permanent solitaire stent placement. Despite residual stenosis, improved distal flow can be seen.

Differentiation of ICAS-LVO from embolic LVO is often challenging, especially in cases without known ICAS and in the setting of EVT for AIS when workups for potential sources of cardioembolism cannot be performed (Figure 1). Several clinical features may be helpful for differentiating ICAS-LVO from embolic LVO (28). Although advanced magnetic resonance imaging (MRI) techniques may provide information on the ischemic zone, thrombus, blood-brain barrier, and vessel wall pathology (29), only non-contrast brain CT and conventional angiographic techniques are available to differentiate these two types of LVOs in most centers.

As shown in Table 1, most investigators used angiographic features for the diagnosis of ICAS-LVO. Baek et al. defined ICAS-LVO as truncal-type occlusion when all major branches and their bifurcation sites are clearly visible beyond the occlusion segment (2, 3). Other investigators considered angiographic findings of residual or fixed stenosis to be ICAS-LVO (4–8).

The prevalence of ICAS-LVO was reported to range from 5.5 to 25%. The prevalence of ICAS in EVT candidates varied depending on the diagnostic methods for ICAS-LVO and race or ethnicity (1, 9).

## Features of ICAS-LVO

ICAS-LVO has more differentiating features than embolic LVO, which are discussed below (Table 2).

**Table 2:** Neuroimaging features and specific considerations in endovascular therapy for large vessel occlusions of intracranial atherosclerotic origin.

**Differential neuroimaging features in ICAS (vs. embolic)**	**Impacts on efficacy and strategies in EVT**	**Specific requirements for ICAS-LVO EVT equipment**	**Assessment tools**
1. Intracranial plaque	Residual stenosis/reocclusion, insufficient expansion of devices, intimal damage, arterial dissection, and vasospasm Long procedure time	Permanent stenting Avoid repeat procedures	HR-MRI Catheter-based imaging (IVUS, OCT)
2. Erythrocyte-poor thrombus	Low recanalization rate with EVT in the presence of fibrin-rich clots Lower recanalization rate with intravenous thrombolysis in ICAS than in embolic stroke Lower recanalization rate with EVT due to reocclusion than red clot	Adjuvant antithrombotics Antiplatelet strategy other than fibrinolytics for *in situ* thrombosis	Thrombus images Pathology of retrieved clot
3. Angioarchitecture			
Calcification and tortuosity	Long procedure time Incomplete recanalization and poor functional outcome	Intermediate catheter^*^	Luminal images Non-contrast CT
Perforator-bearing segment	A higher stroke rate after preventive ICAS intervention	Not available	HR-MRI DWI lesion pattern
Diameter of artery	Increased hemorrhagic complications after preventive ICAS intervention	Intermediate catheter Appropriately sized devices and Solumbra technique^*^	Luminal images
4. Preexisting collaterals	Slower growing and less severe hypoperfusion Higher recanalization rates Better outcome	A longer time window for EVT	Collateral images DWI and PWI pattern
5. Non-atherosclerotic diseases	High restenosis rates in MMD Stent placement may be the preferred treatment in ICAD	Stent placement should be avoided in MMD, but may be considered in ICAD	Detailed clinical and luminal images HR-MRI Catheter-based images

*ICAS, intracranial atherosclerosis; EVT, endovascular therapy; LVO, large vessel occlusion; HR-MRI, high-resolution magnetic resonance imaging; IVUS, intravascular ultrasound; OCT, optical coherence tomography; CT, computed tomography; DWI, diffusion-weighted image; PWI, perfusion-weighted image; MMD, moyamoya disease; ICAD, intracranial arterial dissection*.

* *Theoretical suggestion, not based on the results of clinical studies*.

### Intracranial Plaque

The presence of intracranial plaques can influence endovascular procedures and affect outcome. EVT for ICAS-LVO is associated with residual stenosis or reocclusion, insufficient expansion of devices, inadvertent detachment, arterial dissection, and vasospasm (4, 5, 30). Therefore, repeat procedures and long procedure times are often necessary for successful reperfusion. They are also associated with poor clinical outcomes (2, 6). Repeated stent retrieval attempts, especially in the presence of a plaque at the LVO site, can further damage the fibrous cap and lead to aggravation of *in situ* thrombosis. In western trials, ICAS was less prevalent and early reocclusion after successful reperfusion with EVT was rare (31).

### Perforator

In preventive intervention for ICAS, the incidence of symptomatic complications was high after intracranial stenting for perforator-bearing segments or in patients with branch occlusive disease (BOD) with subcortical infarcts caused by occluding the perforator orifice (32–34). The involved segment was more diffuse and positive remodeling was less frequently observed in BOD-type ICAS than in non-BOD-type ICAS (35, 36). The complication rates of EVT may also be increased in patients with AIS and LVO in the perforator-bearing segments, especially when permanent stent placement is required. Therefore, increased complication rate with the permanent placement of stent in the perforator bearing segment should be considered, especially in the setting of EVT for LVO when appropriate antiplatelet premedication before the procedure is not possible. Further studies are needed because a higher peri-procedural ischemic stroke rate was reported in the treatment of perforator-bearing arteries, and there was no difference between angioplasty alone and balloon mounted/self-expandable stenting (33).

### Arterial Diameter

ICAS-LVO often involves smaller-sized vessels than clots that originated from the heart (such as red clots in atrial fibrillation occluding the distal internal carotid artery). Moreover, the *ring finger protein 213* (*RNF213*) gene variant, the most susceptible gene for moyamoya in Asians, was found in 1 in 4 Japanese and Korean patients with non-moyamoya intracranial stenosis (37, 38). Hongo et al. reported that patients with ICAS and *RNF213* variants had middle cerebral arteries with relatively smaller outer diameter (2.09 ± 0.32 mm) (39). The results of the RCT of the Stenting and Aggressive Medical Management for Preventing Recurrent Stroke in Intracranial Arterial Stenosis showed that treating very small vessels (<2.5–2.75 mm diameter) was associated with higher complication rates, because small vessels are more likely to have restenosis or acute thrombosis and they may also be more prone to injury with stenting (32).

### Calcification And Tortuosity

Patients with ICAS may have stiff, calcified, and tortuous vessels. In these patients, a longer time may be required to reach the target site and incomplete recanalization and poor functional outcomes were reported (40). A *post-hoc* analysis of a RCT showed that the type of intracranial arterial calcification determined the effect of EVT for AIS (41).

### Thrombus

Blood flow affects thrombus composition, with “red clots” or erythrocyte-rich thrombi found in low-pressure systems (heart or venous system), and “white clots” or platelet-rich thrombi found in high pressure systems (e.g., arteries) (42). The composition and burden of clot correlate with revascularization rate in EVT. Fibrin-rich thrombi have higher coefficients of static friction with the vessel walls, and larger thrombi have larger surface areas of thrombus-vessel interaction (43). Treatment response to medical treatment (such as tPA and glycoprotein IIb/IIIa inhibitors) and EVT may vary for ICAS-LVO and embolic occlusion. The thrombus size is usually smaller in ICAS-LVO than in embolic LVO, but the recanalization rates with EVT or tPA were lower in the former than in the latter (43–46). A histopathologic analysis of retrieved thrombi showed that atheromatous gruel (cholesterol clefts, form cells, or fibrous caps) was associated with failed recanalization, and erythrocyte-rich thrombi were associated with successful recanalization (45). In ICAS-LVO cases, adjuvant glycoprotein IIb/IIIa inhibitors for *in situ* thrombosis or angioplasty with/without permanent stent placement may be helpful (2, 3). However, beside stroke subtypes, other factors also influence the characteristics of thrombi, such as collaterals and angioarchitecture (44, 47). In addition, in patients with coronary atherosclerotic plaques, growing thrombi consist of both platelet-rich and erythrocyte-rich clots, and thrombus stability also determines the response to revascularization therapy (48).

### Collaterals

The importance of collateral status has been reported in preventive RCTs of ICAS patients and in acute interventional RCTs (49–52). Although the individual patient data meta-analysis of RCTs of LVO for AIS showed that early treatment with EVT was associated with improved outcomes (53), a recent meta-analysis showed that good collateral status is associated with better clinical responses to EVT even in later time windows, suggesting that collateral status can extend the time window for EVT (54). A retrospective multicenter study of 720 patients showed that while the probability of good outcomes in patients with embolic occlusion declined as onset-to-puncture time increased, the probability of good outcomes in patients with ICAS-LVO did not decline but tended to increase with increase in onset-to-puncture time (8). The incidence of slow progressors may be <30% of patients with anterior circulation LVO in large referral centers (55), but may be higher in ICAS-LVO because collateral circulation in patients with ICAS was better than in those with other stroke subtypes (56).

### Non-atherosclerotic Origin

In addition to ICAS, non-atherosclerotic intracranial arterial diseases, such as moyamoya disease or intracranial arterial dissection, may also cause LVO. Careful evaluation of clinical and luminal studies (such as healthy risk factor profiles and no tandem stenosis or calcification in intracranial arterial dissection, and the presence of family history and basal collaterals in moyamoya disease) may provide clues for the diagnosis of these non-atherosclerotic diseases. However, it is often difficult to differentiate them in clinical practice. Prospective observational high-resolution MRI (HR-MRI) studies of non-stroke subjects (57), young stroke patients (58), and acute stroke patients (59) showed that non-atherosclerotic intracranial large artery disease is prevalent across a wide range of atherosclerosis risk groups. Therapeutic strategies used in intracranial atherosclerosis may not be helpful or may even be detrimental in some patients with non-atherosclerotic LVO (18). For example, stent placement should be avoided in moyamoya disease (60–62), but stent placement (especially, closed cell-type stent) may be considered in intracranial arterial dissection. A recent study showed that endovascular thrombectomy is an effective in selected patients with acute ischemic stroke associated with cervical artery dissection (63), but further studies are needed in patients with acute infarcts due to intracranial non-atherosclerotic occlusion.

## Specific Diagnostic and Therapeutic Considerations in EVT for ICAS-LVO

### Assessment Tools for Underlying Features of ICAS

#### Plaque Images

HR-MRI may provide information on arterial wall pathology, such as plaque characteristics and arterial remodeling. Recently, the imaging findings of intracranial plaques were verified with histopathology (64, 65). A HR-MRI study showed that EVT causes post-recanalization changes of affected arterial segments, which correlated with thrombectomy procedural factors such as number of procedures and type of device used, and was associated with poor outcomes (66, 67). HR-MRI studies conducted after various modes of EVT demonstrated vessel damage related to stentriever process and may be useful for the development of optimal endovascular therapeutic strategies or devices with minimal intimal injury (66, 67). HR-MRI can also provide information on angioarchitecture. Data on the presence and location of perforators in relation to the plaque, in patients with ICAS-LVO, can be useful when considering stent placement in perforator-bearing segments. Lastly, HR-MRI can be used to differentiate non-atherosclerotic intracranial large vessel disease from ICAS in patients. Although concerted efforts have been made to increase signal-to-noise and contrast-to-noise characteristics and to shorten the scanning time, routine use of HR-MRI is not feasible in clinical practice. Like in coronary heart disease, catheter-based imaging can be an alternative modality for use in EVT settings. Intravascular ultrasound (IVUS) and optical coherence tomography (OCT, the light analog of IVUS) are intravascular imaging techniques used in interventional cardiology (68). A meta-analysis of RCTs comparing IVUS- and angiographic-guided percutaneous coronary interventions showed that IVUS guidance was associated with significantly lower rates of angiographic restenosis, repeat revascularization, and overall occurrence of major cardiac events (69). The results of several case reports suggest that these techniques may provide useful information for the selection of patients with ICAS who may benefit from stent placement therapy (16). In addition, IVUS can be used during the EVT procedure to differentiate ICAS-LVO from embolic LVO by visualization of calcified plaque in ICAS. IVUS may help differentiation of intracranial arterial dissection from ICAS and identification of the most distal and proximal extent of arterial dissection, so that the entire length of the dissection could be covered with stent (70). These techniques can also provide virtual histology to characterize plaques in large intracranial vessels. An *in vitro* study of intracranial arterial segments with atherosclerotic plaques demonstrated a strong correlation between virtual histology using IVUS and 7T MRI and histopathologic analysis (71). Gounis et al. recently introduced the high-frequency OCT device for the highly tortuous cerebrovasculature that provides good quality imaging of vessel wall layers, the ostium of small branches/perforators, and the relationship between neurovascular devices and vessel wall (17).

#### Thrombus Images

Identifying the characteristics of a thrombus in AIS may provide vital information for the determination of the optimal strategy for revascularization therapy and for the choice of antithrombotics for the secondary prevention of stroke. The characteristics of a thrombus (size and composition) may determine the recanalization rate, time required for re-opening, and the response to acute and preventive treatment in patients with AIS. Therefore, it is extremely useful to know the thrombus characteristics before initiating recanalization therapy. A thrombus can be detected on a non-contrast CT image as a hyperdense artery sign or a blooming artifact on T2^*^-weighted gradient-recalled image. Details on the methods to measure thrombus size and burden are presented elsewhere (14). Thrombus size determines the response to revascularization. Although thrombus length is strongly associated with successful recanalization with intravenous tPA therapy, the predictive power of thrombus size in determining successful reperfusion in EVT appears to be diminished (14). The results of recent RCTs showed that there was no correlation between the clot burden score (using clot volume and length) and the effects of EVT (72, 73). Thrombus composition and its associated pathogenesis can be visualized by CT or MRI. The density on CT may reflect the thrombus composition. Erythrocytes in thrombi increase attenuation on CT, and the hyperdense artery sign is more commonly seen in erythrocyte-dominant thrombi than in fibrin-rich thrombi. For example, thrombus permeability, as measured by thrombus density on thin-slice non-contrast CT imaging, was found to correlate with the histological components of retrieved thrombi and permeable thrombi were associated with cardioembolic occlusion in patients with AIS (74). However, a recent systematic analysis showed a lack of association between a CT-based clot image (e.g., Hounsfield units) and histopathology of thrombi or stroke etiology (75). Similarly, an erythrocyte component in thrombi induces ferromagnetic field distortion, which results in a blooming artifact on gradient-recalled echo or susceptibility-weighted imaging. The presence of a blooming artifact on MRI is associated with cardioembolic stroke (76, 77). Pathological studies of thrombi retrieved via EVT showed that the presence and absence of blooming artifacts were found to be due to erythrocyte- and fibrin-predominant occlusive thrombi, respectively, and erythrocyte-rich thrombi were associated with successful recanalization of EVT and cardioembolic stroke (78, 79). Lastly, direct thrombus imaging targeting fibrinogen can determine the initial burden and location of thrombi and may also help visualize residual thrombi or distal thromboembolism. Kim et al. investigated hyperacute direct thrombus imaging techniques and monitored the therapeutic efficacy of thrombolysis using fibrin-targeted gold nanoparticles and CT imaging (80). Various MRI probes, such as fibrin-binding gadolinium-labeled peptides, have been used for the evaluation of acute thrombosis after plaque rupture in animal models (81–83).

#### Collateral Images

Conventional angiographic evaluation is the gold standard for collateral assessment (84). However, more time is needed to include the venous phase and contralateral or vertebrobasilar views. In using EVT in clinical settings, most interventionalists perform angiography of the affected territory and open the occluded vessel without performing angiography of unaffected territories in a bid to shorten the puncture-to-reperfusion time. Both multiphase CT angiography and perfusion MRI-based collateral maps can be performed in acute settings (85–87), and they showed a good correlation with conventional angiography for leptomeningeal collateral grading in AIS (87–89). These non-invasive collateral assessments are particularly important in ICAS-LVO, because pre-procedure CT or MRI data can be used for selecting slower infarct progressors presented at a later time. CT angiography shows the anatomical configuration of collateral vessels and its use is becoming more routine. However, there is no consensus on the best method for evaluating and grading collaterals and various CT angiography techniques and grading systems are used (90–92). Other imaging techniques, such as CT perfusion and arterial spin labeling MRI, may also provide information on collateral status (15).

### EVT Devices and Techniques for ICAS-LVO

Stentrievers were the main devices described for use in EVT in the RCTs, and the current guidelines recommend mechanical thrombectomy with a strentriever in conjunction with intravenous tPA as the standard of care in anterior circulation stroke caused by LVOs (93). Owing to the aforementioned characteristics of ICAS-LVO, better tools and techniques are needed for smaller and/or tortuous arteries, the minimization of vessel damage, and the facilitation of rescue therapies. For these purposes, detachable stents with radio-opaque markers for visualizing residual stenosis are required. Stents with radiopaque design can provide better visualization of stent-thrombus interaction during stentrieval process, and also provide additional information on the nature of thrombus as atherosclerotic lesion may appear as an area of strut compression or waist. Permanent stent placement may be required in case of residual stenosis or re-occlusion. In this situation, radiopaque stent strip is informative in stent placement and detachability is essential. However, no radiopaque detachable stentriever is available until now; radiopaque trevo stent is not detachable while solitaire AB is not radiopaque.

In cases in which the relevant artery is tortuous, a large bore balloon guide catheter is preferred, and the stenotic segment of the intracranial artery is crossed with microwire as distally as possible to ensure maximal support while allowing tracking of the balloon guide catheter. To overcome vascular tortuosity, coaxial double-guiding catheter technique, or double-wire technique could be considered (94–96).

In addition, distal access catheters (such as intermediate catheter) provide support and stability for microcatheters and are also suitable for aspiration. The ability to deliver intermediate catheters to the vicinity of the thrombus ensures the generation of greater effective retrieval force by the device especially in cases with significant vessel tortuosity. It also provides a strong enough suction force to remove soft thrombi without using a stent retriever (ADAPT, a direct aspiration first pass technique) (43). Theoretically, this approach is ideal as it results in lesser damage to vessels and underlying plaques, and it may prevent the distal migration of clots to a greater extent than possible with stentrievers. However, the contact aspiration technique requires optimal contact between the aspiration catheter tip and the thrombus, which depends on the location of the thrombus and the tortuosity of the vessel (97). In some cases, the contact aspiration technique may not be effective due to imprecise positioning of the aspiration catheter tip relative to the thrombus. The results of a recent RCT showed no significant differences in the primary outcome of final successful recanalization rates between ADAPT and stentrievers (98). Stentrievers can also be used in conjunction with direct aspiration at the face of a thrombus during thrombectomy (Solumbra technique) (99, 100).

Rescue treatments, including balloon angioplasty, rescue stenting, and intra-arterial glycoprotein IIb/IIIa inhibitor infusion, can be considered for ICAS-LVO refractory to stentriever (3). On the contrary, Yang and the ACUAL investigators studied 302 patients with ICAS-LVO and reported that patients who received angioplasty and/or stenting as first-line therapy showed favorable outcome and lower rate of intracranial bleeding than those received stentriever (10). Further studies are needed in patients with ICAS-LVO to determine the first-line device and technique for thrombectomy (stentriever, ADAPT, or Solumbra), pharmacological adjunct (intravenous tPA or intra-arterial antithrombotics), and cessation time for procedures in cases of repetitive reocclusion.

## Conclusions

Despite the recent success of EVT, there are still numerous challenges with respect to management of ICAS-LVO. Studies discussed herein suggest that there are more diverse neuroimaging features in ICAS-LVO than in embolic occlusion. While recent RCTs of EVT showed that appropriate selection is important in AIS, selection of appropriate EVT procedures may be more important in patients with ICAS-LVO. Patients with ICAS-LVO usually have favorable hemodynamic profiles but demonstrate poor response to EVT. Though ICAS-LVO requires more complex and technically demanding recanalization strategies than embolic occlusion, good outcomes are attainable with the application of appropriate therapeutic strategies.

Future studies should focus on investigating reliable imaging predictors related to response to EVT in ICAS-LVO patients, and on developing and evaluating thrombectomy approaches to overcome the characteristic drawback of reocclusion in ICAS-LVO. Advanced neuroimaging of plaques, thrombi, and collaterals could not be performed in the EVT setting. However, post-EVT analysis may be useful for the characterization of patients with ICAS-LVO, clearer understanding of the pathophysiology of ICAS-LVO, and future guidance for optimal therapeutic strategies for ICAS-LVO. For clinical use of advanced neuroimaging techniques for patients with AIS, fast, and safe assessment tools that can visualize individual features of ICAS, automated software that allows fast post-processing is mandatory, and is increasingly being used in clinical trials (17, 29). In addition, optimal tools and techniques for ICAS-LVO are not settled yet. Most of the studies presented here were retrospective studies conducted in East Asian countries. It is necessary to conduct RCTs of acute interventions for ICAS-LVO in diversified populations to reach recommendations.

## Author Contributions

OB study concept and design, acquisition of data, analysis and interpretation of data, drafting/revising the manuscript for content. BK, W-KS, and PJ drafting/revising the manuscript for content.

### Conflict of Interest Statement

The authors declare that the research was conducted in the absence of any commercial or financial relationships that could be construed as a potential conflict of interest.
